# Supplementary material for: A single mild juvenile TBI in male mice leads to regional brain tissue abnormalities at 12 months of age that correlate with cognitive impairment at the middle age
Source: Acta Neuropathol Commun. 2023 Mar 1;11:32. doi: 10.1186/s40478-023-01515-y (PMC9976423; doi:10.1186/s40478-023-01515-y)
Supplement: Supplementary file 1 — Additional file 1. Table S1. Imaging parameters; Table S2. DTI values and group comparisons from manual DTI analysis; Figure S1. Image analysis process; Figure S2. Decreased vascular diameter is observed in the SI/NB 12m after jmTBI; Figure S3. Correlation between immunolabeling and manual analysis of regional DTI changes; Figure S4. The sucrose preference test was performed at 2 and 12 months. [file 40478_2023_1515_MOESM1_ESM.docx]

**Supplementary Table 1**

|  | ***In vivo* MRI** | |
| --- | --- | --- |
| **Seq. Type** | T2 (Bruker: MSME) | DTI (Bruker: DtiEpi) |
| **TR (ms)** | 3290 | 1000 |
| **TE (ms)** | 7 | 30 |
| **NEX** | 2 | 1 |
| **FOV (mm)** | 16x12.8 | 16x12.8 |
| **Matrix** | 162X128 | 162x128 |
| **Slices (n)** | 24 | 48 |
| **Slice Thickness (mm)** | 0.533 | 0.266 |
| **Slice Interval (mm)** | 0 | 0 |
| **Echos** | 25 | 1 |
| **Flip Angle** | 0 | 90 |
| **Resolution (mm)** | 0.098 x 0.1 | 0.098 x 0.1 |
| **Number of directions** | n.a. | 21 |

**Supplementary table 1. Imaging parameters.**

| **DTI** | **ROI** | | **stats** | **sham** | **jmTBI** |  | **DTI** | **ROI** | | **stats** | **sham** | **jmTBI** |
| --- | --- | --- | --- | --- | --- | --- | --- | --- | --- | --- | --- | --- |
| **FA** | **DG** | **ipsi** | mean | 0,2800 | 0,2466 |  | **RD** | **DG** | **ipsi** | mean | 0,3487 | 0,3785 |
|  |  |  | p* |  | **0,0477** |  |  |  |  | p* |  | 0,4524 |
|  |  | **contra** | mean | 0,2754 | 0,2705 |  |  |  | **contra** | mean | 0,3582 | 0,3761 |
|  |  |  | p* |  | 0,9635 |  |  |  |  | p* |  | 0,8014 |
|  | **CA1** | **ipsi** | mean | 0,2389 | 0,2589 |  |  | **CA1** | **ipsi** | mean | 0,4569 | 0,4138 |
|  |  |  | p* |  | 0,2818 |  |  |  |  | p* |  | 0,2104 |
|  |  | **contra** | mean | 0,2552 | 0,2419 |  |  |  | **contra** | mean | 0,4295 | 0,4544 |
|  |  |  | p* |  | 0,7665 |  |  |  |  | p* |  | 0,6567 |
|  | **SI/NB** | **ipsi** | mean | 0,2468 | 0,2008 |  |  | **SI/NB** | **ipsi** | mean | 0,2889 | 0,2639 |
|  |  |  | p* |  | 0,2526 |  |  |  |  | p* |  | 0,5322 |
|  |  | **contra** | mean | 0,2575 | 0,2424 |  |  |  | **contra** | mean | 0,2603 | 0,2580 |
|  |  |  | p* |  | 0,8477 |  |  |  |  | p* |  | 0,9943 |
| **AD** | **DG** | **ipsi** | mean | 0,5459 | 0,5544 |  | **MD** | **DG** | **ipsi** | mean | 0,4145 | 0,4371 |
|  |  |  | p* |  | 0,9377 |  |  |  |  | p* |  | 0,6241 |
|  |  | **contra** | mean | 0,5572 | 0,5735 |  |  |  | **contra** | mean | 0,4245 | 0,4419 |
|  |  |  | p* |  | 0,7459 |  |  |  |  | p* |  | 0,7797 |
|  | **CA1** | **ipsi** | mean | 0,6328 | 0,5976 |  |  | **CA1** | **ipsi** | mean | 0,5155 | 0,4751 |
|  |  |  | p* |  | 0,3589 |  |  |  |  | p* |  | 0,2486 |
|  |  | **contra** | mean | 0,6125 | 0,6290 |  |  |  | **contra** | mean | 0,4905 | 0,5126 |
|  |  |  | p* |  | 0,7424 |  |  |  |  | p* |  | 0,6724 |
|  | **SI/NB** | **ipsi** | mean | 0,4236 | 0,3604 |  |  | **SI/NB** | **ipsi** | mean | 0,3338 | 0,2961 |
|  |  |  | p* |  | **0,0294** |  |  |  |  | p* |  | 0,2219 |
|  |  | **contra** | mean | 0,3919 | 0,3798 |  |  |  | **contra** | mean | 0,3042 | 0,2986 |
|  |  |  | p* |  | 0,8440 |  |  |  |  | p* |  | 0,9638 |

**Supplementary table 2. DTI values and group comparisons from manual DTI analysis.** Significant differences are higlighted in red and in bold. FA_fractional anisotropy, AD_axial diffussivity, RD_radial diffussivity, MD_mean diffussivity. *p-values correspond to Sidak post-hoc test following Two-way ANOVA, sham vs jmTBI.


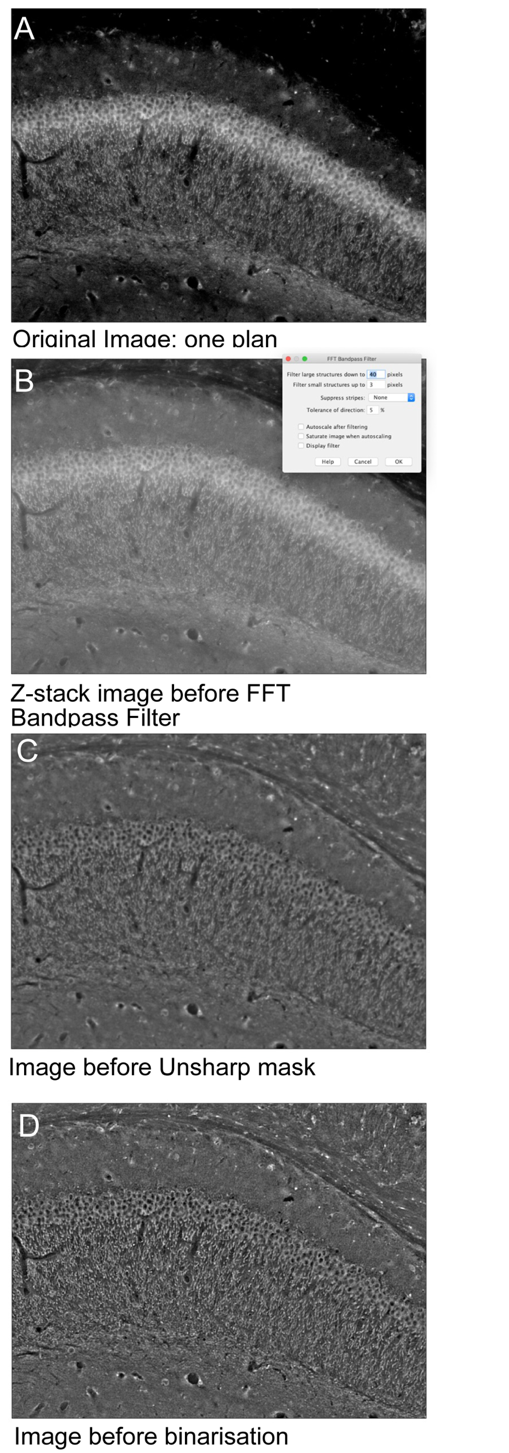


**Supplementary figure 1.** Image analysis process

Using Z-stack function in FIJI software, stack images (A) were reconstructed in one plane (B). Then, reconstructed image was treated with “Bandpass” filter without autoscale after filtering (C). The result image was treated with “Unsharp mask” (D) and then binarised.

**
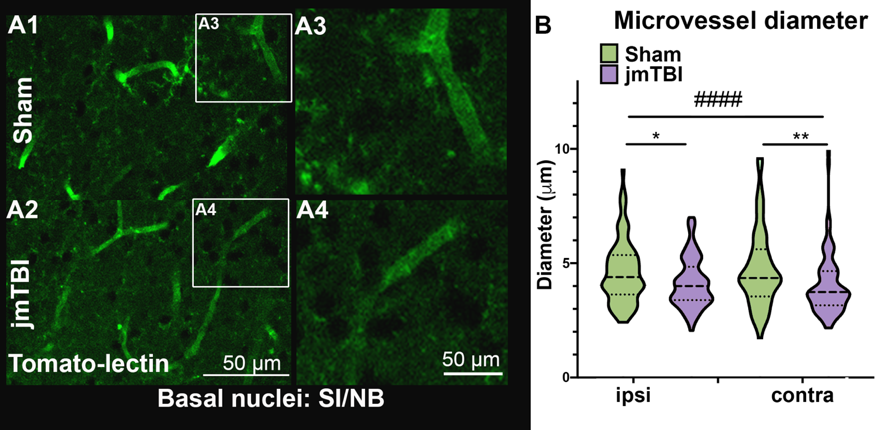
**

**Supplementary figure 2. Decreased vascular diameter is observed in the SI/NB 12m after jmTBI.**

The reduction in vessel diameter in jmTBI compared to sham mice was significant in both ipsilateral and contralateral sides of the SI/NB. Two-way ANOVA (# indicating global jmTBI vs sham difference) with Sidack post-hoc test (* indicating jmTBI vs sham differences in the ipsilateral or contralateral side). Data expressed as mean+SEM. **P<0.01, ####P<0.0001

**
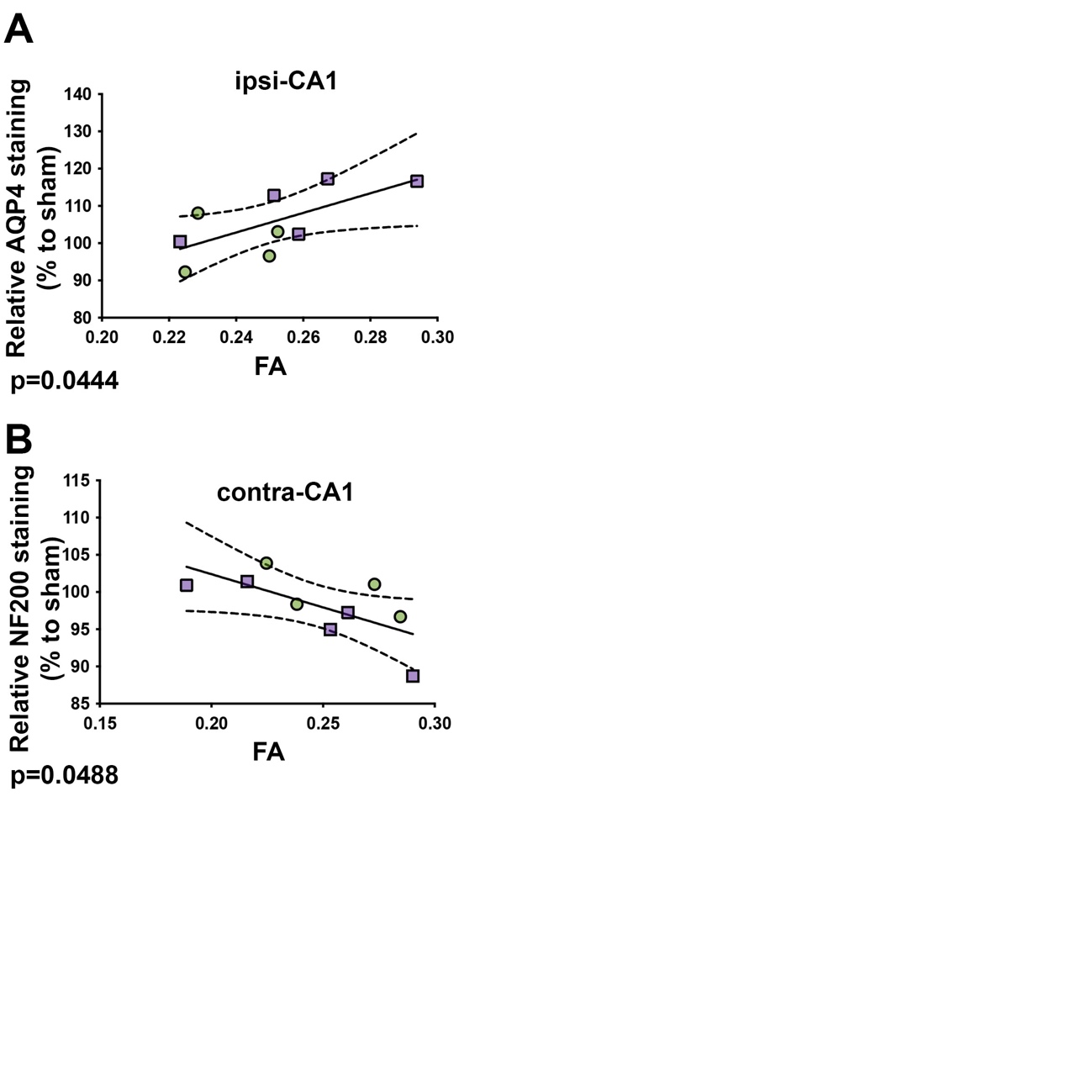
**

**Supplementary figure 3. Correlation between immunolabeling and manual analysis of regional DTI changes**


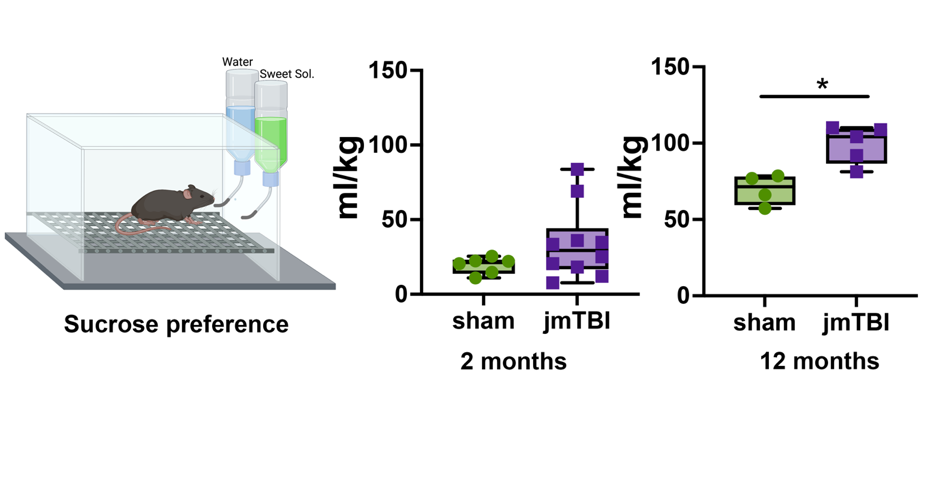


**Supplementary figure 4.** The sucrose preference test was performed at 2 and 12 months. A significant increase in sucose consumption was observed at 12 months compared to the sham (p<0.05).Two-way ANOVA with Sidak post-hoc test. Data expressed as mean+SEM. *P<0.05
